# Children with Cerebral Palsy and Their Parents Have Different Experiences of Pain Management: A Qualitative Study

**DOI:** 10.3390/children11091055

**Published:** 2024-08-29

**Authors:** Elisabeth Rønning Rinde, Agneta Anderzén-Carlsson, Reidun Birgitta Jahnsen, Randi Dovland Andersen

**Affiliations:** 1 Division of Habilitation, Telemark Hospital Trust, 3710 Skien, Norway; 2 Research Center for Habilitation and Rehabilitation Models & Services (CHARM), Department of Public Health, Institute of Health and Society, Faculty of Medicine, University of Oslo, 0373 Oslo, Norway; reijah@ous-hf.no (R.B.J.); anrd@sthf.no (R.D.A.); 3 University Health Care Research Centre, Faculty of Medicine and Health, Örebro University, 701 82 Örebro, Sweden; agneta.anderzen-carlsson@regionorebrolan.se; 4 Faculty of Health and Social Sciences, Inland Norway University of Applied Sciences, 2406 Elverum, Norway; 5 NorCP, Department of Clinical Neurosciences for Children, Oslo University Hospital, 0450 Oslo, Norway; 6 Department of Research, Telemark Hospital Trust, 3710 Skien, Norway

**Keywords:** childhood cerebral palsy, childhood pain, child vs. parent experiences, lived experience, pain management, parenting

## Abstract

**Aim**: The aim of this study is to explore and compare experiences of pain management strategies for children with CP from the perspectives of children themselves and their parents. **Methods**: A secondary inductive analysis of previously collected qualitative data was performed. Fourteen children with CP and one parent of each child were interviewed separately about the management of the child’s pain. A dyadic data analysis was used to compare parents’ and children’s perspectives. **Findings**: The main thematic categories of pain management identified were self-care, psychological strategies, physical interventions and professional treatment. Experiences described by the child and parent differed within all participating dyads but to different degrees. On a group level, children described more use of psychological strategies than parents did. Parents described more professional treatment strategies. **Conclusions**: Parents and children described different experiences of pain management strategies, and both perspectives are needed to understand the child’s situation.

## 1. Introduction

Children with cerebral palsy (CP) have a high risk of experiencing pain [1,2]. CP encompasses a group of permanent movement disorders caused by a lesion in the immature brain [3]. The clinical picture varies greatly and depends on the extent, location and timing of the injury [3]. Pain may occur as a secondary complication caused by primary consequences of the brain injury (for example spasticity, muscle contractures and joint malalignment) or from treatment procedures like stretching, injections and surgery. Consequently, children with CP often experience procedural pain, other acute pain episodes and chronic pain [4,5].

Pain in children with CP is often undertreated [6,7]. The best way to achieve pain relief is through an individually tailored combination of prevention, physical, psychological and pharmacological strategies, also referred to as a 4P approach [8]. The optimal combination for each child is highly individual and depends upon a multitude of factors, some of which may include the type of pain [4,9], the child’s personality, cognitive function, previous experience and family culture [10]. Consequently, advice on pain management strategies should be individually tailored based on information about the child and family [11].

As pain is a personal experience [12], only the person living with pain can know exactly how it feels and what may provide pain relief [13]. However, a proxy report of pain is necessary for very young children and for children who cannot self-report their pain [14,15] either verbally or by using augmentative or supplementary communication strategies. Parents of children with CP feel responsible for their children’s pain management [16,17], and clinicians often ask parents to provide information about their child, including information about pain and pain management [18]. However, to the best of our knowledge, there are no studies investigating the correspondence between children’s and their parents’ descriptions of what helps with pain—neither for children in general nor for children with CP.

Correspondence between children’s and parents’ evaluation of the child’s pain, on the other hand, has been the topic of several studies addressing pain in children with CP. While some studies found that parental assessments of pain in children with CP corresponded with that of the child [18,19], other studies have identified differences [1,20]. Parkinson and colleagues [1] found that parents tended to overestimate mild and infrequent pain but underestimated more severe and frequent pain. Despite inconclusive results, studies have shown that children’s and parents’ evaluation of the child’s pain sometimes differ.

Factors related to both child and parent may explain the differences between them in evaluating the child’s pain. Only the child experiences the pain, while the parent evaluates the pain based on observations of the child’s verbal communication, gestures and behavior [10]. Children may deliberately or unintentionally over- or under-communicate their pain [21,22]. They may underreport pain to avoid a hospital visit they are afraid of or overreport pain to avoid school or make others notice their pain. Factors such as gender, personality and previous experience of observing others in pain have been found to influence our evaluation of others’ pain [23], and parents’ evaluation of their child’s pain is found to be affected by their own experiences of pain [24]. Furthermore, in a qualitative interview study, parents of children with CP described difficulties in evaluating their child’s pain, caused by the child’s unwillingness to speak of it [17].

Since an assessment of pain is a prerequisite for deciding upon the management of pain and evaluation of pain management interventions [11], it is reasonable to assume that children’s and parents’ experiences of pain management may also sometimes differ. However, this issue has not yet been addressed in the scientific literature.

To improve the management of pain in children with CP, we need more in-depth knowledge about if and how parents’ and children’s experiences of pain management may differ and correspond. Moreover, such knowledge can make us more able to meet and understand the individual child in the clinical context.

## 2. Aim of the Study

The aim is to explore and compare experiences of pain management in children with CP from the perspectives of children themselves and their parents.

## 3. Materials and Methods

### Design

This qualitative multifamily-member study [25] with an inductive descriptive design [26] comprised a secondary analysis of data collected for the purpose of exploring experiences of pain from the perspectives of children and adolescents with CP and their parents. Children’s and parents’ individual experiences of pain in general have been described in previous publications [blinded for review].

## 4. Procedures

### 4.1. Sample and Setting

Fourteen child–parent dyads were recruited from two pediatric rehabilitation centers in southern Norway and included based on demographic criteria of the child. The inclusion criteria were age between 8 and 17 years with a CP diagnosis, ability to communicate verbally with or without communication support and having experienced pain during the last week or on regular basis. To capture as much variation in experiences as possible, a purposive sampling strategy [26] was used to include children of varying age and gender and, with different degrees of motor and cognitive (dys)function.

Families with a child who met the inclusion criteria were first approached by a clinician and informed about the project. Those who agreed to participate were contacted by the first author and given more information to secure informed consent.

Five girls and nine boys aged between 9 and 16 years (mean 12.9 years) were included. Based on information from parents, the children’s motor function according to the Gross Motor Function Classification System (GMFCS) [27] ranged from level I to level V, with a majority of nine children at level I (walking without assistance). Their cognitive and communicative difficulties ranged from none to moderate. Six children received special education. One child was diagnosed with moderate intellectual disability and two with autism. Thirteen mothers and one father participated. The age range for parents was 36–50 years (mean 41 years). Nine children lived with both parents, four had parents who lived apart with shared custody and one parent had sole custody.

### 4.2. Data Collection

Data were collected in semi-structured individual interviews [26] conducted by the first author. In all but one dyad, the parents were interviewed first. Interview guides were used to ensure that topics related to the research questions were covered, but participants were encouraged to speak freely about topics they found relevant. Since we wanted children and parents to talk about their own experiences in their own words, the questions were open-ended. They were followed up with more detailed questions when necessary. The interview guides thus functioned as checklists, helping to guide the interviews into the following topics: the experience of having pain, how the pain influenced everyday life (activities, participation) and pain management (both self-mastery and others’ support). The topics were the same for children and parents, but parents were also asked about the consequences of the child’s pain for both the child and themselves. For children who had cognitive or communicative challenges, visual support was used to facilitate the interviewing, and all participating children were able to describe their experiences. The data addressing children’s and parents’ experiences of what helped to manage pain in the children were used in the current study.

### 4.3. Data Analysis

The method for analyzing multifamily-member interviews outlined by Van Parys [25] inspired the data analysis in this study. The aim of the analysis was to identify similarities and differences between descriptions by child and parent in each dyad and across all dyads. The first step was to inductively analyze both interviews from a dyad separately and code all citations about pain management on a descriptive and interpretative level.

A short summary of the content covered by the codes in each interview was written. The initial coding of each of the interviews resulted in a set of preliminary thematic codes, representing different pain management strategies. In the second step, data coded to these preliminary thematic codes (i.e., pain management strategies) were compared within each dyad to identify similarities and differences in how the child and their parent described pain management. The preliminary thematic codes were discussed among all authors and revised to form codes that described all the data. These were further grouped into meaningful themes on a more abstract level of content. The codes formed subthemes under each of these themes. The third and final step was to identify similarities, or common themes, and differences across all dyads. Tables with themes and subthemes from parents and children in each dyad were used to facilitate this step of the analysis.

The first author did the initial coding, but between the second and third step and again after the third step, interpretations and coding were discussed among all authors to increase trustworthiness. The group of authors consisted of one physiotherapist with extensive clinical and research experience regarding people with CP over the life course, one pediatric nurse with clinical experience from working with children with various disabilities and with extensive experience within qualitative pediatric and disability research, one registered nurse with clinical experience from working with neonates and experience from research on pain in both children with and without disabilities and one neuropsychologist with clinical experience from the pediatric rehabilitation field.

### 4.4. Ethical Considerations

All participants received age- and developmental-appropriate written and verbal information about the study. Parents and children > 16 years provided written informed consent to participate in the research and towards publication of results. Parents consented on behalf or younger children. In addition, the children themselves gave their verbal assent.

The study was approved by the Norwegian Committee for Medical and Health Research Ethics (REC South-East, 46,124) 3 February 2020 and the Data Protection Official for Research at Telemark Hospital (19/02788) 3 April 2020 after a review by the Norwegian Centre for Research Data (571,250). To protect privacy, demographic information is given on group level (children and parents separately) instead of linked in the dyads. In addition, each parent and child was interviewed separately, and the information they provided was treated confidentially within the dyad.

## 5. Findings

The themes ultimately identified in the coding process of all data were self-care, psychological approaches, physical strategies and professional treatment. Themes with corresponding subthemes are presented in Table 1.

In all dyads, the child and parent differed in their description of useful pain management strategies. Their differences encompassed all four main themes. Still, some dyads were more coherent in their responses than others were.

Across all dyads, some differences between children and parents were identified on a group level. Children described using more psychological strategies than the parents, while parents described the use of more professional treatment strategies than the children did. Findings will be presented in narrative form under the headings of each identified theme. A more comprehensive presentation of the results is provided in Table 2, illustrating the detailed coding of individual children’s and parent’s experiences.

## 6. Self-Care

Self-care included activities children themselves or their parents initiated to reduce pain. Strategies like resting, getting enough sleep, drinking enough water and facilitating defecation were examples of self-care used to prevent or manage pain. Resting was mentioned by nearly all participants and therefore overlapped in most dyads. When it came to other types of self-care, children and their parents tended to mention different strategies. For example, some children found it helpful to move a little when they experienced pain, but only one parent mentioned this. The experience of activity limitation for pain prevention also differed within most dyads and was most often mentioned by the parent only.

## 7. Psychological Strategies

Psychological strategies included passive and active distraction and cognitive strategies involving a deliberate change in thought to manage pain. There was a lack of consistency within the dyads regarding the use of psychological strategies to manage pain. The children generally mentioned using psychological strategies to a higher degree than their parents did. The only psychological strategies mentioned by parents were distraction, comfort and emotional support. The following quotes from one dyad illustrate how both child and parent mentioned passive distraction (use of iPad), while the child also described other psychological strategies (active distraction and cognitive strategies):

“I knock on the table many times and then I cuddle Teddy, my teddy bear which is a big teddy bear. (…) I sit with the iPad, and then sometimes I get a little bit… then I walk around the room and think, a little bit and go ‘what if it doesn’t go well’ and stuff, if I can’t calm down.” # Child 1

“She gets a little quiet, and then there’s this thing about ‘I want iPad’ (…) she sits down with the iPad and then she relaxes, and disappears all the way into the iPad.” # Parent 1

Doing something enjoyable was a strategy mentioned by several children but by one parent only. Children also described several other beneficial strategies, such as thinking good thoughts, seeking knowledge about pain, seeking peer support and “think the pain out of the body”, none of which was mentioned by parents. Consequently, the child’s and parent’s experiences of active distraction and cognitive strategies differed within almost all dyads.

Comfort and emotional support included hugs, words of encouragement and parents being present when the child suffered pain. Although mentioned by several parents and children, it was only reported by both child and parent within one of the dyads. Consequently, parents and children within most dyads expressed different experiences of the usefulness of comfort and emotional support.

## 8. Physical Strategies

Physical strategies described to relieve pain were stretching, exercise and massage. The experience of stretching overlapped within dyads and was mentioned by both the child and their parent. The experience of massage, on the other hand, differed within dyads. Both children and parents mentioned its use, but there was consistency in experience of its benefit within only three dyads. Furthermore, parents mentioned it more often than the children did.

### Professional Treatment

The treatment theme included strategies involving professional health care, either in consultations or through prescriptions and the adaptation of aids and medication. In general, the experience of professional treatment strategies differed within most dyads. Professional treatment strategies for alleviating pain, such as physiotherapy or chiropractic therapy, were primarily mentioned by parents. The injection of muscle relaxants and orthopedic surgery were mentioned by two children and their parents and by an additional two parents. Pain medication was mentioned by more parents than children. Orthoses and a standing frame were mentioned by an equal number of parents and children but only one child and parent in the same dyad.

## 9. Discussion

Within all participating dyads, the child and their parent described different experiences of pain management strategies. On a group level, the children generally described using more psychological strategies than their parents did, and only parents mentioned the use of professional treatment strategies like physiotherapy and chiropractic therapy. These two themes differed most in the participating dyads, but differences occurred within all themes. In almost all dyads, the child and parent agreed that rest was an effective strategy but differed in reporting about other self-care strategies. On a group level, children and parents mentioned about the same number of physical interventions, but in most dyads, the child and parent mentioned different physical intervention strategies. One of the reasons for these differences may be that children and parents understand the child’s pain differently [20,28]. For example, it is possible that children initiate self-care measures or psychological strategies they perceive as useful without parents registering that their child is experiencing pain. Consequently, the parent will not recognize the child’s activity as a pain management strategy.

The participants in this study described a wide variety of strategies used to manage pain. In accordance with current best practices, these strategies covered prevention as well as psychological, physical and pharmacological strategies, the so-called 3P or 4P approach, depending on whether or not prevention is included [8]. Activity limitation was frequently mentioned, and physical overload is found to increase the risk of pain for children with CP [9,29]. Based on the results from this study, it seems reasonable to suggest that activity limitation as a way to prevent pain could be considered an important pain management strategy for this population.

In this study, children described a wider repertoire of psychological strategies than their parents. This may indicate that parents were not aware of the children’s use of such strategies. If parents are unaware that their child uses psychological strategies and finds them efficacious, this is of great clinical importance and may indicate a need for more pain education for parents. Psychological strategies like active and passive distraction, guided imagery, breathing exercises and memory reframing are found to be useful for children in general [14]. Despite the fact that children in our study described the use of psychological strategies, children with CP have been found to use fewer psychological strategies than their peers without CP and also to start using them later in life [30]. Increased knowledge of the effectiveness and use of psychological strategies can help children gain a wider repertoire of psychological strategies and use them more effectively and at an earlier age. Pain education for parents could enable them to help their children to develop such strategies. Since studies indicate that pain in childhood CP is undertreated [6,7], health professionals may also need more competence in the area of psychological strategies in order to provide pain education for children and parents on the topic.

There was considerable variation among the participating families regarding the degree of correspondence between the child’s and parent’s description of how to prevent or manage pain. Different degrees of correspondence may be caused by different family cultures for dealing with pain [10,31]. When parents have a high consciousness about pain, this may result in better correspondence between child and parent because pain is often discussed. Children may feel supported when parents and others understand their pain [32,33], and this may indicate that a highly developed common understanding of pain and pain management between parent and child is preferable. However, high consciousness about pain in the family may also increase the child’s pain experience [34] since the experience and expression of pain is formed in interaction with others [35]. Parents can, on the other hand, be balancing support and helping their child to cope with pain by not giving it too much attention [17]. This may result in lower correspondence between the child’s and parent’s description because pain is less discussed in the family. However, based on the data used in this analysis, it is not possible to say why the degree of correspondence differed in the participating dyads.

Children and parents described comfort and emotional support differently. In some dyads, only the parent mentioned it. This raises the question of whether or not the child found these strategies helpful. One explanation may be that children take comfort from parents for granted and do not “label it” as pain management. This latter interpretation is supported by experiences of children with leukemia who described managing treatment-related strain better when parents were close by and emotionally engaged in their situation, without being able to elaborate on the exact mechanism for this support [36]. In other dyads, only the child mentioned comfort and emotional support. This indicates that some children find these measures helpful even if their parent perceives them as a self-evident part of parenting or are not fully aware of the possible pain-relieving effect of comfort and support. Parents of children with CP often feel responsible for their child’s pain management [16] and may feel powerless when their child suffers from pain [17]. This may threaten the parent’s own psychological health, whereas the feeling of competence seems to reduce this negative effect [37,38]. Parents’ lack of awareness regarding how their behaviors and actions may influence their child’s experience of pain may be a contributor to this felt helplessness. If health professionals through pain education help parents understand the importance of providing comfort and support in managing their child’s pain, parents may feel more able to help and feel less ineffectual. Differences between children and parents in the description of comfort and emotional support may also be due to different perceptions of what it means that something “helps”. While some may have interpreted it narrowly as something that directly affects the pain, others may have interpreted it more broadly and included anything that makes someone feel better. The first interpretation would probably not include comfort, while the second interpretation might do so.

On a group level, children and parents in this study described many strategies, but the individual child and parent seemed to use a limited repertoire of strategies compared to what is described in the literature. This may be because they had considered other strategies to be less useful, but it may also mean that there were relevant strategies they were not familiar with. A study indicating that children with CP appeared to be self-taught when it came to cognitive strategies [39] may point in that direction. If potentially useful strategies are unknown in some families, there is a potential for improved pain management through pain education [11]. Health professionals could contribute by adding a broader range of strategies and increase understanding of strategies that appeared to be underutilized. Training could also address the combined use of different types of strategies (physical, psychological, pharmacological and preventive strategies) and how they collectively yield a better outcome than using individual strategies or a more random composition of strategies [8,14].

Several differences between children and parents with potential clinical relevance were identified. Only the child experiences the pain, and children in this study described several self-initiated strategies their parents did not seem to be aware of. On the other hand, young children in particular may have less overview and understanding than parents do. This is substantiated by the fact that none of the children in our study mentioned professional treatment, such as muscle relaxant injections, as helpful even though these interventions have been found to reduce pain [40]. Our results indicate that children and parents have complementary knowledge about pain management strategies, and therefore, both perspectives need to be included in clinical practice.

## 10. Strengths and Limitations

The key strengths of this study are its novelty and the participant-centered approach. To the best of our knowledge, no other studies have compared the experiences of pain management strategies in children with CP and their parents. Since previous knowledge in this area was currently lacking, a qualitative exploratory approach was considered the most appropriate.

The participants were encouraged to speak freely about what helped against pain. They were free to focus on what they considered important related to pain management. However, since no systematic questions were asked regarding pain management strategies not mentioned by the participant, some differences between children and parents may be due to individual variations in what came to mind during the interview rather than real differences in their perception of what helped relieve pain. For example, some of the children might have confirmed that muscle relaxants help against pain if they had been asked about this directly. Further multi-method research is needed to determine the coherence between children’s and parents’ experiences with regard to what alleviates pain in children with CP.

## 11. Conclusions

Children and parents described different yet complementary experiences of helpful pain management strategies. Professionals need to elicit and include both perspectives: first, to gain a more complete understanding of the child’s pain situation and, second, to leverage this knowledge in discussions with the child and parents to help manage the child’s pain in the best way possible.

## Figures and Tables

**Table 1:** Overview of themes and subthemes.

Theme	Subtheme
Self-care	Rest
	Stay warm
	Sleep, food, water
	Movement, positioning
	Light, daily exercise
	Facilitate defecation
	Activity limitation
Psychological Strategies	Passive distraction
	Active distraction
	Cognitive strategies
	Seek knowledge of pain
	Seek peer support
	Comfort and emotional support
Physical strategies	Stretching
	Massage
	Exercise
Professional treatment	Physiotherapy, chiropractic therapy
	Use of orthoses, standing frame
	Muscle relaxant injections, surgery
	Pain medication

**Table 2:** Overview of findings. Red color illustrates the number of parent-child dyads were only the child mentioned the pain-management strategy in question. The blue illustrate that only the parent in the dyad mentioned the strategy, whereas the purple color illustrate that both child and parent In the dyad mentioned the strategy.

Dyads	1	2	3	4	5	6	7	8	9	10	11	12	13	14
Self-care														
Rest														
Activity limitation														
Stay warm (bath, sauna)														
Movement, positioning														
Sleep, food, water														
Light daily exercise														
Facilitate defecation														
Psychological strategies														
Comfort and emotional support														
Passive distraction														
Active distraction														
Cognitive strategies														
Seek knowledge of pain														
Seek peer support														
Physical interventions														
Stretching														
Massage														
Exercise														
Treatment														
Pain medication														
Use of orthoses/standing frame														
BoNT-A injections/Surgery														
Physiotherapy/Chiropractic														
Data from 14 dyads.														
Purple = agreement within dyad														
Red = mentioned by child only														
Blue = mentioned by parent only														

## Data Availability

Data are contained within the article.

## References

[1] Parkinson K.N., Dickinson H.O., Arnaud C., Lyons A., Colver A., Beckung E., Parkes J., Fauconnier J., Michelsen S., on behalf of the SPARCLE group (2013). Pain in young people aged 13 to 17 years with cerebral palsy: Cross-sectional, multicentre European study. Arch. Dis. Child.

[2] Mckinnon C.T., Meehan E.M., Harvey A.R., Antolovich G.C., Morgan P.E. (2019). Prevalence and characteristics of pain in children and young adults with cerebral palsy: A systematic review. Dev. Med. Child Neurol..

[3] Rosenbaum P., Paneth N., Leviton A., Goldstein M., Bax M., Damiano D., Dan B., Jacobsson B. (2007). A report: The definition and classification of cerebral palsy April 2006. Dev. Med. Child Neurol..

[4] Hauer J., Houtrow A.J. (2017). Pain Assessment and Treatment in Children With Significant Impairment of the Central Nervous System. Pediatrics.

[5] Ramstad K., Jahnsen R., Skjeldal O.H., Diseth T.H. (2011). Characteristics of recurrent musculoskeletal pain in children with cerebral palsy aged 8 to 18 years. Dev. Med. Child Neurol..

[6] Westbom L., Rimstedt A., Nordmark E. (2017). Assessments of pain in children and adolescents with cerebral palsy: A retrospective population-based registry study. Dev. Med. Child Neurol..

[7] Sultan T., Wong C. (2023). Presence and grade of undertreatment of pain in children with cerebral palsy. Scand. J. Pain.

[8] Taddio A., Appleton M., Bortolussi R., Chambers C., Dubey V., Halperin S., Hanrahan A., Ipp M., Lockett D., MacDonald N. (2010). Reducing the pain of childhood vaccination: An evidence-based clinical practice guideline (summary). Can. Med Assoc. J..

[9] Ostojic K., Paget S.P., Morrow A.M. (2019). Management of pain in children and adolescents with cerebral palsy: A systematic review. Dev. Med. Child Neurol..

[10] Craig K.D. (2020). A child in pain: A psychologist’s perspective on changing priorities in scientific understanding and clinical care. Paediatr. Neonatal Pain.

[11] Trottier E.D., Ali S., Doré-Bergeron M.-J., Chauvin-Kimoff L. (2022). Best practices in pain assessment and management for children. Paediatr. Child Health.

[12] Raja S.N., Carr D.B., Cohen M., Finnerup N.B., Flor H., Gibson S., Keefe F.J., Mogil J.S., Ringkamp M., Sluka K.A. (2020). The revised International Association for the Study of Pain definition of pain: Concepts, challenges, and compromises. Pain.

[13] Fillingim R.B. (2017). Individual differences in pain: Understanding the mosaic that makes pain personal. Pain.

[14] Eccleston C., Fisher E., Howard R.F., Slater R., Forgeron P., Palermo T.M., Birnie K.A., Anderson B.J., Chambers C.T., Crombez G. (2021). Delivering transformative action in paediatric pain: A Lancet Child & Adolescent Health Commission. Lancet Child Adolesc. Health.

[15] Belew J.L., Barney C.C., Schwantes S.A., Tibboel D., Valkenburg A.J., Symons F.J., McGrath P.J., Stevens B.J., Walker S.M., Zempsky W.T. (2013). Pain in children with intellectual or developmental disabilities. Oxford Textbook of Paediatric Pain.

[16] Ståhle-Öberg L., Fjellman-Wiklund A. (2009). Parents’ experience of pain in children with cerebral palsy and multiple disabilities—An interview study. Adv. Physiother..

[17] Rinde E.R., Anderzén-Carlsson A., Jahnsen R.B., Andersen R.D. (2023). “Pain is one piece of a complex jigsaw puzzle”—Experiences of raising a child with cerebral palsy who has pain. Disabil. Rehabil..

[18] Hägglund G., Burman-Rimstedt A., Czuba T., Alriksson-Schmidt A.I. (2020). Self-versus Proxy-Reported Pain in Children with Cerebral Palsy: A Population-Based Registry Study of 3783 Children. J. Prim. Care Community Health.

[19] Özcan F., Delialioğlu S., Özel S., Demir Y. (2022). Perception of pain in patients with adolescent cerebral palsy: Self report or parent’s report. Somatosens. Mot. Res..

[20] Büğüşan S., Kahraman A., Elbasan B., Mutlu A. (2018). Do adolescents with cerebral palsy agree with their caregivers on their participation and quality of life?. Disabil. Health J..

[21] Boerner K.E., Chambers C.T., Craig K.D., Riddell R.R.P., Parker J.A. (2013). Caregiver accuracy in detecting deception in facial expressions of pain in children. Pain.

[22] Larochette A.-C., Chambers C.T., Craig K.D. (2006). Genuine, suppressed and faked facial expressions of pain in children. Pain.

[23] Ruben M.A., Hall J.A. (2013). “I know your pain”: Proximal and distal predictors of pain detection accuracy. Pers. Soc. Psychol. Bull..

[24] Ndengeyingoma A., Lebel V., Alvarez S.B. (2023). Children and pain: Assessment and management according to parents’ perspective. Res. Nurs. Health.

[25] Van Parys H., Provoost V., De Sutter P., Pennings G., Buysse A. (2017). Multi family member interview studies: A focus on data analysis. J. Fam. Ther..

[26] Patton M.Q. (2015). Qualitative Research & Evaluation Methods: Integrating Theory and Practice.

[27] Rosenbaum P.L., Palisano R.J., Bartlett D.J., Galuppi B.E., Russell D.J. (2008). Development of the Gross Motor Function Classification System for cerebral palsy. Dev. Med. Child Neurol..

[28] Kamper S.J., Dissing K.B., Hestbaek L. (2016). Whose pain is it anyway? Comparability of pain reports from children and their parents. Chiropr. Man. Therap.

[29] Brunton L.K., Bartlett D.J. (2013). The bodily experience of cerebral palsy: A journey to self-awareness. Disabil. Rehabil..

[30] Chaleat-Valayer E., Roumenoff F., Bard-Pondarre R., Ganne C., Verdun S., Lucet A., Bernard J.C. (2019). Pain coping strategies in children with cerebral palsy. Dev. Med. Child Neurol..

[31] Palermo T.M., Valrie C.R., Karlson C.W. (2014). Family and parent influences on pediatric chronic pain: A developmental perspective. Am. Psychol..

[32] Castle K., Imms C., Howie L. (2007). Being in pain: A phenomenological study of young people with cerebral palsy. Dev. Med. Child Neurol..

[33] Rinde E.R., Anderzén-Carlsson A., Jahnsen R.B., Andersen R.D. (2023). “I have to obey my pain”—children’s experiences of pain burden in cerebral palsy. Disabil. Rehabil..

[34] Chambers C.T., Craig K.D., Bennett S.M. (2002). The impact of maternal behavior on children’s pain experiences: An experimental analysis. J. Pediatr. Psychol..

[35] Craig K.D. (2009). The social communication model of pain. Can. Psychol./Psychol. Can..

[36] Leibring I., Anderzen-Carlsson A. (2022). Young children’s experiences of support when fearful during treatment for acute lymphoblastic leukaemia-A longitudinal interview study. Nurs. Open.

[37] Glenn S., Cunningham C., Poole H., Reeves D., Weindling M. (2009). Maternal parenting stress and its correlates in families with a young child with cerebral palsy. Child Care Health Dev..

[38] Skok A., Harvey D., Reddihough D. (2006). Perceived stress, perceived social support, and wellbeing among mothers of school-aged children with cerebral palsy. J. Intellect. Dev. Disabil..

[39] McKinnon C.T., White J.H., Morgan P.E., Antolovich G.C., Clancy C.H., Fahey M.C., Harvey A.R. (2020). The lived experience of chronic pain and dyskinesia in children and adolescents with cerebral palsy. BMC Pediatr..

[40] Ramstad K., Jahnsen R., Lofterod B., Skjeldal O.H. (2010). Continuous intrathecal baclofen therapy in children with cerebral palsy—When does improvement emerge?. Acta Paediatr..

